# Skeletal age-at-death estimation: validating the Suchey-Brooks method using 3D reconstructed models in a contemporary Indonesian population

**DOI:** 10.1007/s00414-025-03496-0

**Published:** 2025-04-21

**Authors:** Ridhwan Lye, Zuzana Obertová, Nur Amelia Bachtiar, Daniel Franklin

**Affiliations:** 1 Centre for Forensic Anthropology, M420, The University of Western Australia, Crawley, WA 6009 Australia; 2 Radiology Department, Hasanuddin University, Jalan Perintis Kemerdekaan KM. 10, Talamanrea, Makassar 90254 Indonesia

**Keywords:** Forensic anthropology, Age estimation, Pubic symphysis, Transition analysis, Bayesian statistics, Indonesia

## Abstract

The Suchey-Brooks (S-B) standard is one of the most frequently applied approaches for age-at-death estimation in modern forensic practice. However, classification accuracy is known to vary across different populations. At present, there is a paucity of research related to the assessment of biological attributes in Indonesia, particularly the estimation of age-at-death. The use of computed tomography (CT) in S-B phase assignments has been validated in the literature. In considering further validating the use of CT, transition analysis (TA), and Bayesian statistics in age-at-death estimation, this study evaluated the accuracy of the S-B standard on a sample obtained from Indonesia. TA and Bayesian statistics are incorporated to address methodological issues such as age mimicry. A total of 378 multi-slice CT scans were analysed in *OsiriX*^®^. TA and Bayesian statistics were used to derive age-at-death estimation models. Overall bias values were at − 6.0 years for females and − 13.1 years for males, while inaccuracy was at 9.6 years for females and 14.6 years for males. When applying the original S-B age ranges, 92.0% of females and 73.3% of males were correctly classified. Likewise, mean ages per S-B phase were higher in the Indonesian sample, except for females assigned to Phase VI. TA and Bayesian statistics derived age-at-death distribution models specific to the Indonesian population. The dissemination of an appropriate age-at-death estimation standard in the literature is of considerable benefit to casework conducted domestically in Indonesia, and also serves to further inform aspects of general forensic practice globally.

## Introduction

The pubic symphyseal face is the most researched region in the human skeleton for the estimation of age-at-death [1–6], with the standard developed by Brooks and Suchey [2] (S-B) the most preferred by forensic practitioners [7, 8]. The S-B standard, developed in a population sample from the United States, assesses several features of the symphyseal face by assigning one of six pre-defined phases [2]. Researchers have noted that differences in ageing trajectories can be explained by variations in nutrition, genetics, and environmental factors [9], and that age-at-death estimates may become less accurate the further removed an unknown individual is from the reference sample [10].

The S-B standard has been tested in population groups from Australia and Southeast Asia. In the former, Lottering et al. [11] reported correct classifications of 63.9% of females and 69.7% of males with Queensland-specific age-at-death distribution models developed using transition analysis (TA) and Bayesian statistics were used. Similarly poor results were reported in a Victorian sample, with 67.1% of females and 57.6% of males correctly assigned to within 1SD of their respective S-B phase (see Merritt [12]). For the latter, in a Thai sample, Schmitt [13] reported 37.9% of females and 36.1% of males were correctly assigned within 1SD value of their respective S-B phases. Much higher values were reported in a Malaysian population, in which 97.89% of females and 96.36% of males were correctly assigned (see Hisham et al. [14]). The variance in classification performance in these studies underscores the importance of deriving population-specific models for age-at-death estimation, although other methodological issues likely contribute to those variances, such as age mimicry.

Traditional age-at-death estimation standards, including Brooks and Suchey [2], are likely to introduce bias through age mimicry [15, 16]. The mean and confidence intervals of age-at-death estimates are derived directly from the distribution of the sample used. This may skew their values to fit the sample rather than fit the population group being tested. In some cases, the distribution of the sample is skewed toward younger individuals (e.g., less than 40 years, in [4]). Consequently, the age estimates derived from such skewed distributions would decrease when attempting to estimate age in older individuals (i.e., 40 + years) [16].

TA and Bayesian statistics can be applied to attempt to address of age mimicry. TA involves the use of a generalised linear model to estimate the probability of an S-B phase being associated with an individual of a specific age [15]. Researchers have compiled data from various population groups into bespoke computer programs, such as ADBOU and TA3, to aid forensic practitioners in estimating age-at-death due to the computational complexity of performing Bayesian analyses [8, 16]. However, these programs deviate from the S-B standard by analysing specific features on the symphyseal face and scoring them as individual components rather than as a whole. By instead focusing on the morphological changes in the symphyseal face as a whole, the S-B standard is significantly easier to use and is less time-consuming [2].

In countries where physical reference skeletal collections are rare or otherwise do not exist, virtual anthropology has been identified as a viable proxy to physical skeletal material, with various studies in the literature validating its use with the S-B standard [11, 14, 17]. Virtual anthropology also facilitates acquisition of contemporary samples [10]. With Indonesia having recorded a high frequency of mass fatality events throughout its history, the development of Indonesia-specific forensic anthropological standards is paramount for practitioners who may rely on its use in routine medicolegal casework and instances of disaster victim identification [18]. As such, the aims of the present study are to evaluate the accuracy and reliability of the S-B standard as applied to an Indonesian population using pelvic CT scans, and to derive population-specific age-at-death estimation models using TA and Bayesian statistics.

## Materials and methods

### Study sample

**Fig. 1:**
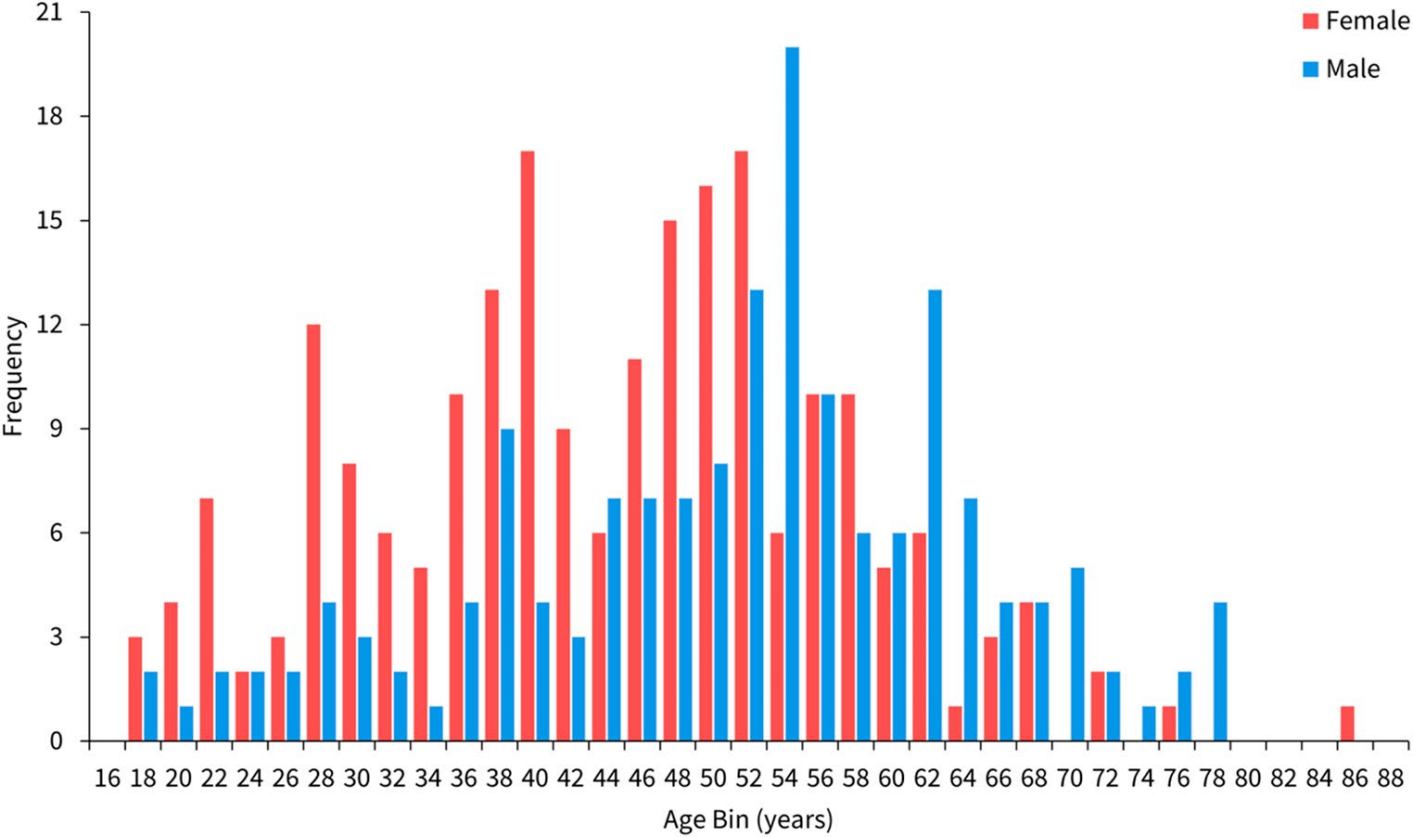
Age distribution of the sample used in this study, sorted by sex

A total of 378 multi-slice CT (MSCT) scans were obtained from Dr Wahidin Sudiohusodo National General Hospital (RSWS) within Hasanuddin University in Makassar, Indonesia. The study sample comprised 213 females (mean age = 43.7 years; SD = 12.8 years) and 165 males (mean age = 50.9 years; SD = 13.3 years). Their ages ranged from 17 to 86 years. The age and sex distribution of the study sample is provided in Fig. 1.

The scans were acquired from the Picture Archiving and Communications System database in RSWS, representing patients who presented to the hospital for radiological examination as part of their routine medical treatment between January 2020 and August 2022. All patient data, except recorded age and sex at the time of scanning, were anonymised prior to receipt.

A Siemens Healthineers SOMATOM go.Top 128-slice was used to obtain the scans with slice thickness between 1.0 and 1.5 mm (95.5% of all scans were 1.5 mm). Any MSCT scans that displayed signs of pathology, or any other abnormality that would obscure the viewing and assessment of the pubic symphyseal face, were excluded from this study at the sample collection stage.

Collection and analysis of the sample was approved by the Office of the Director-General of Health Sciences from the Ministry of Health, Republic of Indonesia, through Hasanuddin University (LB.02.01/2.2/6807/2022). Approval to conduct this study was granted by the Human Ethics Committee of the Office of Research at the University of Western Australia (2021/ET000377), in accordance with the Australian National Statement on Ethical Conduct in Human Research.

### Visualisation and S-B phase assignment

All MSCT scans were visualised using *OsiriX*^®^*v13.0.1* using the ‘3D volume rendering’ function. Specifically, the ‘High Contrast’ 3D preset was used, with the CLUT set to ‘VR Muscles-Bones’. No convolutional filters were applied. The ‘3D rotate’ and ‘pan’ functions were used to orientate the scans into its respective view. To remove any unwanted structures, such as medical devices or other non-relevant anatomical regions, the scissor tool was used. Once each scan was properly orientated, phases were assigned to both the left and right pubic symphyseal face according to the written descriptions and illustrations provided by Brooks and Suchey [2], supplemented by physical cast specimens manufactured by *France Casting*^®^.

### Statistical analysis

Non-Bayesian statistics detailed in this study were performed using *IBM SPSS Statistics v29.0.0*. TA and Bayesian statistics were performed on *R v4.4.0* with *RStudio v2024.04.0* using scripts from Prof. Lyle Konigsberg [19] that were modified for the purpose of this study.

#### Intra-observer agreement

Intra-observer agreement is quantified using a subset of 50 randomly selected MSCT scans comprising 25 female (mean age = 48.9 years; SD = 16.7 years) and 25 male scans (mean age = 49.4 years; SD = 15.4 years). These scans were assessed by RL three times, with each attempt performed with an interval of at least 24 h. Only the left symphyseal face was assessed. Agreement was calculated using the intraclass correlation statistic (ICC). The interpretation of ICC values is as follows [20]: poor when < 0.50, moderate when between 0.50 and 0.75, good when between 0.75 and 0.90, and excellent when > 0.90.

#### Bilateral asymmetry

Previous studies have noted that bilateral asymmetry may affect phase assignments between the left and right symphyseal faces in the same individual [11, 13, 14]. A Wilcoxon signed-rank test ($\:Z$) was used to assess bilateral asymmetry in the present sample.

#### Bias, inaccuracy, and classification accuracy of S-B phase assignments

Age-at-death estimation models often include measures of reliability to evaluate the performance of the S-B standard when applied across population groups. Within the context of this study, reliability is measured using two approaches: bias and inaccuracy [21]. Bias represents the mean degree of over- or under-estimation of age in years,

$$\:\frac{\sum\:({A}_{est}-{A}_{rec})}{n}$$

while inaccuracy is the absolute error of age estimation in years,

$$\:\frac{\sum\:|{A}_{est}-{A}_{rec}|}{n}$$

where $\:{A}_{est}$ is the estimated age, $\:{A}_{rec}$ is the recorded age, and $\:n$ is the sample size [22]. Specifically, $\:{A}_{est}$ is associated with the mean age of the S-B phase assigned to the individual.

Performance of the S-B standard as applied to the Indonesian sample is also evaluated according to classification accuracy (i.e., the percentage of individuals whose recorded age falls within the unmodified age range of their assigned S-B phase). Any individuals whose recorded age falls outside of that range would be classified as either being assigned to an S-B phase above or below (i.e., categorical misclassification as opposed to bias).

Mean age values between the original published values in the S-B standard and those aggregated by phase assignments in the Indonesian sample are compared against each other using two-tailed paired-sample *t*-tests; one for females and one for males. To assess whether the distribution of S-B phases is significantly different between females and males, a Mann-Whitney U test was performed.

#### Transition analysis and bayesian statistics

TA and Bayesian statistics were used to model the age-at-death distributions for the Indonesian population. Models were derived for both Indonesian females and males. To build these models, TA was first performed using a log-age cumulative probit analysis to obtain age-at-transition parameters [23]. These models assess the average age an individual transitions from one S-B phase to the next by calculating a series of intercept and common slope parameters that are then transformed to mean age-at-transition and standard deviation (SD) values [15, 16, 24]. Since age is scaled logarithmically in this model, the resulting age-at-transition distributions do not include negative age values when plotted on a normal age scale.

Once TA is performed, Bayesian statistics are then used to obtain age-at-death probabilities. Specifically, the probability of an individual being of a specific age conditional on the observed S-B phase. Bayes theorem is expressed mathematically as follows [11, 25]:

$$\:\text{P}\text{r}\left(a|{c}_{j}\right)=\frac{\text{P}\text{r}\left({c}_{j}|a\right)f\left(a\right)}{\underset{0}{\overset{\infty\:}{\int\:}}\text{Pr}\left({c}_{j}|x\right)f\left(x\right)\text{d}x}$$

where $\:\text{P}\text{r}\left(a\right|{c}_{j})$ is the probability of an individual at age $\:a$, given the observed S-B phase, $\:{c}_{j}$. $\:\text{P}\text{r}\left({c}_{j}\right|a)$ is the probability of an observed S-B phase given the age of the individual obtained though TA. $\:f\left(a\right)$ is the probability density function for age [26] and generally includes the use of an informative prior (e.g., mortality data) fitted to a hazard model (e.g., Gompertz-Makeham or Kaplan-Meier) [27]. Since mortality data for the Indonesian population are not available to use as an informative prior, a uniform prior was used. Such prior assumes that any individual is equally likely to die at any age across their lifespan– in the context of this study, from age 17 to 86 years [16].

Combining both TA and Bayesian statistics, age-at-death distribution plots (i.e., the posterior density regions) were modelled for each S-B phase. The highest posterior density (HPD) intervals at 50% and 95%, and point estimates using maximum likelihood, are provided [17, 28].

## Results

### Intra-observer agreement and bilateral asymmetry

The ICC estimate and 95% confidence interval (CI) for intra-observer agreement on phase assignment was based on a single-rating, absolute-agreement, 2-way mixed-effects model. Phase assignments across the three attempts had moderate agreement, $\eqalign{&\:\text{I}\text{C}\text{C}=0.750,\:F\left(98,\:98\right)\cr&=9.93,\:p<.001,\:95\%\:\text{C}\text{I}\:[0.637,\:0.839].\cr}$

For bilateral asymmetry, the Wilcoxon signed-rank test indicated no significant differences in phase assignments from the left and right symphyseal faces, $\:Z=0.64,\:p=.521$. Of 378 pairs, 68.5% ($\:n=259$) were a match between the left and right faces. All non-matched pairs had differences of one S-B phase maximum (e.g., the left symphyseal face was assigned Phase II while the right was assigned Phase III). Consequently, phase assignments from the left symphyseal face were used for all further statistical analyses.

### Changes in pubic symphysis morphology

Descriptive statistics for each S-B phase assigned to the Indonesian sample are provided in Table 1. The distribution of S-B phase assignments between females and males were significantly different, $\:U=2.38,\:p=.017$. From these distributions, females had a lower mean age value per S-B phase compared to males. In general, the mean age between females and males increases from one S-B phase to the next, with smaller increments observed between Phases I and III (1.2 and 3.0 years, respectively), to larger increments between Phases V and VI (8.8 and 8.0 years, respectively). The standard deviation values in the Indonesian sample are greater than those reported in the original study [2]. The small sample size for males in Phases I and II ($\:n=5$) suggest that interpreting the mean age values should be performed with caution.

**Table 1:** Descriptive statistics for the S-B phases as assigned in Indonesian males and females. All age values are presented in years

Phase	*n*	Mean Age	SD	SE	95% CI	Range
Female						
I	16	21.3	3.0	0.8	19.8–22.7	17–27
II	24	34.0	10.3	2.1	29.9–38.1	21–60
III	44	43.9	11.0	1.7	40.6–47.1	25–71
IV	59	46.0	11.3	1.5	43.1–48.9	28–86
V	57	49.1	9.0	1.2	46.7–51.4	35–68
VI	13	54.7	9.2	2.5	49.7–59.7	44–75
Male						
I	5	22.4	5.6	2.5	17.5–27.3	17–30
II	5	28.2	15.1	6.8	14.9–41.5	19–55
III	30	46.9	10.9	1.9	43.2–50.7	24–68
IV	66	49.7	10.4	1.3	47.2–52.2	28–77
V	49	57.9	11.5	1.6	54.0–61.1	30–78
VI	10	62.7	7.2	2.3	58.2–67.2	51–71

### Bias, inaccuracy, and classification accuracy of S-B phase assignments

Bias and inaccuracy values sorted by chronological age brackets are presented in Table 2. Bias values for females ranged from − 34.0 to 0.7 years, and males − 27.1 to 2.0 years. Inaccuracy values ranged from 3.3 to 34.0 years for females and from 8.4 to 16.0 years for males. In general, females have lower bias and inaccuracy values than males. S-B phase assignments were also more accurate in females than males for younger individuals (i.e., ≤ 49 years). The ages of all individuals aged 50 years and older were under-estimated. The inaccuracy value of 34.0 years for females aged 70–84 years should be interpreted with caution due to a small sample ($\:n=4$).

**Table 2:** Bias and inaccuracy values for phase assignments in the Indonesian sample, compared against the S-B standard, sorted by known ages. All age values are presented in years

Known Age	Female			Male		
	*n*	Bias	Inaccuracy	*n*	Bias	Inaccuracy
17–29	33	0.0	3.3	13	2.0	8.4
30–39	49	0.7	6.9	22	–1.1	15.3
40–49	59	–3.8	8.1	30	–10.0	16.0
50–59	50	–11.8	12.6	55	–16.3	11.5
60–69	18	–19.7	19.7	32	–19.2	11.1
70–84	4	–34.0	34.0	13	–27.1	10.5
Total	213	–6.0	9.6	165	–13.1	14.6

The age estimates using the S-B standard were accurate in 84.4% of the Indonesian sample (Table 3). Specifically, 92.0% of females and 74.5% of males were correctly assigned. Misclassifications occurred in all six phases; however, these were all over-estimations and only by one phase (i.e., their recorded age was lower than the age range of the assigned S-B phase).

**Table 3:** Classification accuracies for S-B phase assignments in the Indonesian sample

Phase	S-B Age Range		Correct Phase		Diff. Phase^a^		Total	
	Female	Male	*n*	%	*n*	%	*n*	%
I	15–24	15–23	16	4.2	3	0.8	19	5.0
II	19–40	19–34	23	6.1	3	0.8	26	6.9
III	21–53	21–46	50	13.2	5	1.3	55	14.6
IV	26–70	23–57	110	29.1	20	5.3	130	34.4
V	25–83	27–66	96	25.7	15	4.0	112	29.6
VI	42–87	34–86	23	6.1	13	3.4	36	9.5
Total			319	84.4	59	15.6	378	100.0

^a^ All differences in the study sample were over-estimations by one S-B phase

**Fig. 2:**
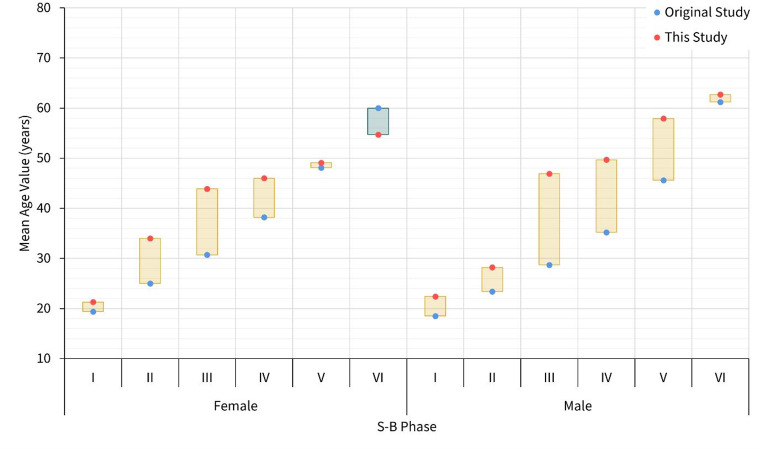
Mean age differences between the original S-B standard and this study, sorted by sex. The orange bars indicate higher mean ages in this study, while the blue bar indicates a lower mean age

Mean age differences between Brooks and Suchey [2] and the present study are described in Fig. 2. The mean age values were greater for every phase in Indonesian males and females compared to those reported in Brooks and Suchey [2], except for Phase VI for females. The differences were greater than 10 years in Phase III for females (by 13.2 years), and in Phases III to V in males (by 18.2, 14.5, and 12.3 years, respectively). While these differences were significant in males, $\:t\left(5\right)=3.36,\:p=.02$, there was no statistically significant difference in females, $\:t\left(5\right)=1.69,\:p=.151$.

### Transition analysis and bayesian statistics

**Fig. 3:**
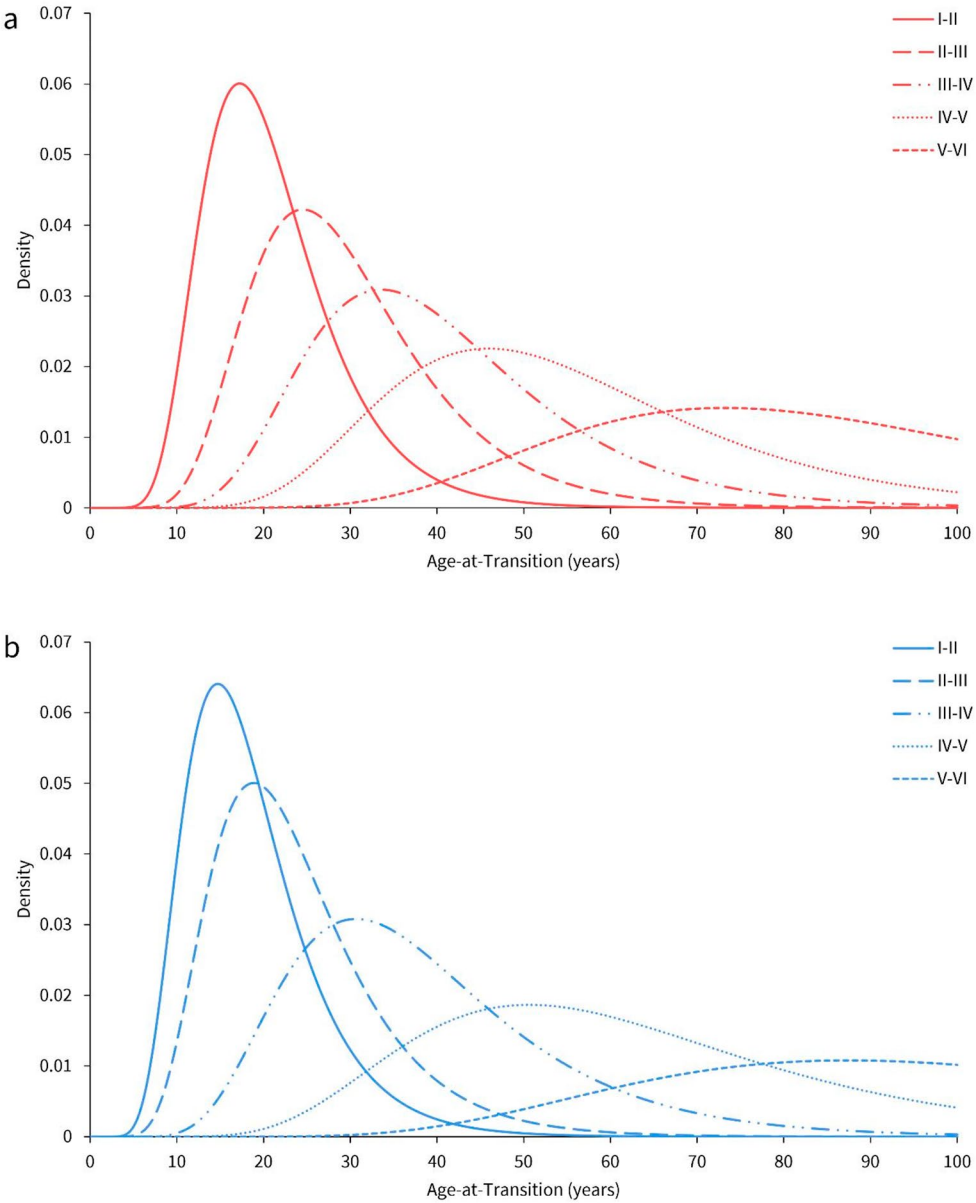
Log-normal age-at-transition distribution plots between each successive S-B phase in (**a**) females and (**b**) males, derived from the log-age cumulative probit model

Age-at-transition plots for both females and males are detailed in Fig. 3. The horizontal axis for age was converted from log-age to the normal scale. Mean log age-at-transition (estimate) and standard error (SE) for each transition state, along with the common sex-specific SD values, are provided in Table 4. The SD values indicate the degree of variability in mean age estimates in each transition state. Figure 3 shows that each transition state is distinct, although the variability within each is large. The overlap between successive transition states is larger in males than in females. Females were also observed to begin transitioning between S-B phases earlier than males.

**Table 4:** Descriptive statistics associated with each age-at-transition state for the Indonesian sample, sorted by sex. Age values for exp(Est) are presented in years

Parameter	Female			Male		
	Estimate	SE	exp(Est)^a^	Estimate	SE	exp(Est)
I–II	2.98	0.97	19.7	2.84	1.16	17.2
II–III	3.33	1.02	27.9	3.09	1.20	22.0
III–IV	3.64	1.05	38.2	3.58	1.25	35.8
IV–V	3.96	1.06	52.3	4.08	1.25	59.0
V–VI	4.42	1.09	83.3	4.62	1.32	101.9
SD	0.36	0.28	–	0.39	0.32	–

^a^ exp(Est) = maximum likelihood estimate of age-at-transition

**Fig. 4:**
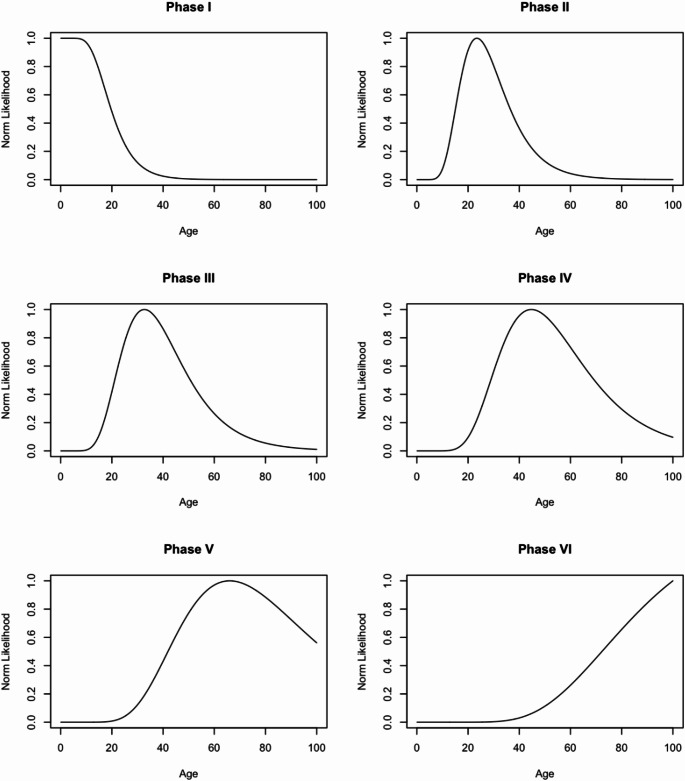
Normed likelihood distribution plots for females in the Indonesian population derived from the log-age transition analysis and uniform prior, sorted by S-B phase

**Fig. 5:**
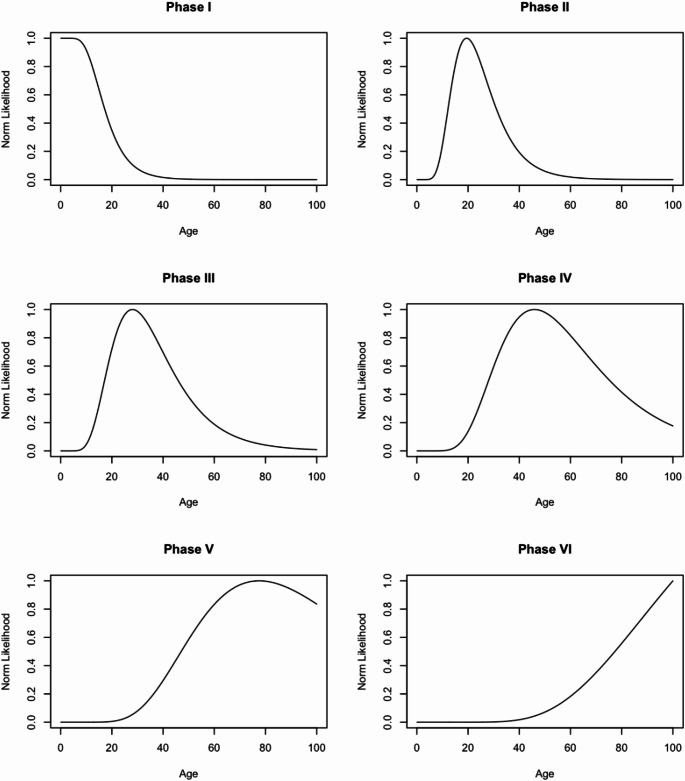
Normed likelihood distribution plots for males in the Indonesian population derived from the log-age transition analysis and uniform prior, sorted by S-B phase

Normed likelihood distributions for each S-B phase in Indonesian females and males are visualised in Figs. 4 and 5, respectively. In the distribution plots, the uniform prior is used, scaled to the maximum likelihood value of 1.0. Point estimates, 50%, and 95% HPD intervals derived from these distributions are shown in Table 5. Phases I and VI for both female and male distributions have truncated ages at 17 and 86 years, respectively, relative to methodological considerations. Compared against the frequentist approach, regardless of sex, the mean ages in Phases I to IV were lower than the point estimate ages in the Bayesian approach. In contrast, the point estimate ages in Phases V and VI were higher than the frequentist values.

**Table 5:** Pubic symphyseal age-at-death estimations, including point estimate and highest posterior density indices. Age values are presented in years

Phase	Point Est	50% HPD^a^		95% HPD	
		Lower End	Upper End	Lower End	Upper End
Female					
I	17.0	17.0	22.1	17.0	36.8
II	23.5	18.5	29.7	17.0	50.8
III	32.6	25.2	42.3	17.0	66.5
IV	44.7	34.6	57.8	22.6	86.0
V	66.0	50.2	86.0	30.9	86.0
VI	86.0	82.6	86.0	53.4	86.0
Male					
I	17.0	17.0	21.6	17.0	36.1
II	19.4	19.4	25.6	17.0	45.8
III	28.1	21.1	37.3	17.0	64.1
IV	46.1	34.0	62.4	20.6	86.0
V	77.5	57.5	86.0	35.0	86.0
VI	86.0	85.0	86.0	56.8	86.0

^a^ HPD = highest posterior density

## Discussion

The aims of the present study were to evaluate the performance of age-at-death estimates using the S-B standard and to derive age-at-death probability distribution models specific to the contemporary Indonesian population. Results from this study indicate an acceptable level of accuracy (i.e., above 80.0%) for the assignment of S-B phases to the Indonesian sample against their recorded age. However, all mean age values, except for females in Phase VI, were lower in the Indonesian sample compared to those published in Brooks and Suchey [2]. However, these differences were only significant in the male subset.

### Intra-observer agreement and bilateral asymmetry

Intra-observer agreement in this study was moderate ($\:\text{I}\text{C}\text{C}=0.750$), which is consistent with previous research testing the S-B method, particularly in CT scans [11, 14, 29, 30]. Intra-observer agreement is the more appropriate statistic to measure for this study as a low agreement value would preclude any meaningful assessment of inter-observer agreement. Likewise, inter-observer agreement was not considered in this study as past research has already demonstrated acceptable agreement values between observers [14, 30, 31]. As such, the use of virtual samples as a proxy to physical material in estimating age-at-death using the S-B standard has been further validated.

Bilateral asymmetry in phase assignment was not significant in the present study. The original S-B standard did not explicitly indicate a preferred side for the assessment of the pubic symphyseal face, although the authors provided visualisations taken from the left side in their publication [2]. One study reported a significant effect of bilateral asymmetry in their population [11], while another noted no significant effect [14]. However, most studies report results from one symphyseal face (i.e., either the left or right face) [29, 30] or do not specify which side was used for phase assignment [17, 28]. It is recommended that phase assignments should be made on the left side, as implicitly directed in the S-B standard, unless the left innominate is missing or otherwise damaged.

### Reliability of the S-B standard

The application of the S-B standard across population groups highlights the differences in mean ages within each phase [e.g., 14, 30] and/or their associated age-at-death models [e.g., 11, 28]. The morphology of the pubic symphyseal face of individuals of the same chronological age between population groups can be influenced by a variety of factors, including diet and nutrition, hormones, and the environment [32]. Amongst other factors, it has been noted that population groups that exhibit larger body sizes (e.g., taller and/or greater body mass index) appear to transition between S-B phases later than those which exhibit a smaller body size [33]. This difference would therefore result in the S-B standard overestimating age-at-death in larger individuals and underestimating it in smaller individuals [34].

The S-B standard accurately assigned 84.4% of all individuals in the Indonesian sample. This is lower than in a Malaysian population, where 97.18% of their sample were correctly assigned [14]. These values, however, are still higher than in other studies using CT scans for S-B age estimation. Lottering et al. [11] reported accuracy values of 63.9% with the left symphyseal face and 69.7% with the right symphyseal face in a Queensland population. Wink [35] reported an accuracy of 79.5% in a US sample, although the sample analysed was small ($\:n=44$).

Overall, the S-B standard is more accurate in Indonesian females, with an overall bias of − 6.0 years and inaccuracy of 9.6 years. The standard is also more accurate with individuals aged below 40 in the Indonesian sample, with bias values as low as 0.0 years and inaccuracy values of 3.3 years. These values, along with the classification accuracies presented, are similar to other studies that report the same statistics [14, 36, 37]. The same research also highlights the decreasing reliability of the S-B standard in estimating age-at-death for individuals aged 40 years and older, with bias and inaccuracy values as large as − 17.93 and 18.39 years, respectively [14]. Such values relative to individuals younger than 40 years, and a greater number of misclassifications reported in this study, support this assertion.

The inclusion of 3D representations of each S-B phase in this study was not considered. Previous studies assessing the validity of the S-B standard with digital modalities have demonstrated comparable gross morphological similarities with physical skeletal material and have included them in their publications [14, 17, 29]. Furthermore, other population-specific models for age-at-death estimation using the S-B standard do not include 3D representations for their respective phases [11, 28].

### TA and bayesian statistics

Traditional age-at-death estimation models use a frequentist approach, wherein the age range and confidence interval values associated with each S-B phase are used to indicate whether an unknown individual’s age-at-death does or does not fall within those specified values. In contrast, TA and Bayesian statistics provide probability values associated with the age-at-death estimate according to the assigned S-B phase [17]. In practice, forensic anthropologists would be able to stipulate that an unknown male individual with an assigned S-B phase of III would have their ‘true’ age-at-death fall within the range of 21 to 37 years with a probability of 50%. In considering other age-at-death estimation standards from the Southeast Asian region, this study is one of the first to incorporate both TA and Bayesian statistics in relation to the S-B standard. One other Southeast Asian study testing the S-B standard only included TA [14]. Without the addition of Bayesian statistics, only age-at-transition intervals can be used, which is inaccurate as these values do not measure the same variable (i.e., estimating age-at-transition, not age-at-death).

Incorporating an informative prior for the derivation of age-at-death distributions was not considered, as mortality data for Indonesia is not available. Consequently, a uniform prior was used. These priors are typically considered more conservative [17, 28], but still serve towards negating the effects of age mimicry that often affect the reliability and/or accuracy of age estimation standards [38]. However, these distributions should be interpreted with caution, as the upper bounds for the later S-B phases, particularly in Phases V and VI, may have point estimates and HPD values that are calculated to be extremely large (see Figs. 4 and 5). Other studies which incorporate a uniform prior also report similarly large upper bound age values [17, 28].

The age-at-death distribution plots, regardless of whether a uniform or informative prior is used, indicate broader variability in symphyseal degeneration within population groups [11, 17, 28]. Despite these issues, the symphyseal face is still recommended as the preferred skeletal region for the derivation of age-at-death estimates [21]. However, researchers have suggested utilising a component-based scoring system [15, 16], similar to McKern and Stewart [4]. Computer software used for age-at-death estimation, including ADBOU and TA3, use component scorings for their data entry [8, 16].

### Study limitations & future research

The MSCT scans analysed in this study had a slice thickness that is larger than many other studies in the literature [11, 14, 17]. The features observed in the pubic symphyseal face require high detail to be reliably quantified; the larger the slice thickness (i.e., lower resolution), the more detail is lost. This does not appear to have been an issue in the present study, as evidenced by appropriate levels of intra-observer agreement. Further, similar to the present study, Savall et al. [30] analysed CT scans with a slice thickness of 1.5 mm, which was deemed sufficient for visualising all pertinent features in the pubic symphyseal face. Hall et al. [28] assessed the applicability of CT as a viable alternative to physical material and noted that a CT slice thickness of 2.0 mm was too large to facilitate visualisation of features in the pubic symphyseal face. It would thus appear that 1.5 mm slice thickness is the minimum requirement for research of this nature.

This study sampled individuals from Makassar, one of many population centres in Indonesia. Its applicability to the broader Indonesian population has not yet been tested. Future research should consider validating this standard in other Indonesian sub-populations to further improve its accuracy and maintain statistical robusticity. Such attempts have already been made in other countries with large, disparate population centres, such as Australia [11, 39].

## Conclusion

The present study has demonstrated reduced accuracy of the S-B standard when applied to an Indonesian population. Population-specific models derived in the present study provide forensic practitioners in Indonesia access to a reliable and accurate skeletal age-at-death estimation standard for use in both routine and DVI casework. The incorporation of TA and Bayesian statistics facilitated reporting of probability values associated with an age-at-death estimate and address issues of age mimicry. CT scans as a viable alternative to physical skeletal material has also been further validated by this study. With the addition of this standard in the forensic anthropological literature, practitioners may consider its applicability in other Indonesian sub-populations to further improve its accuracy and applicability.
